# Thin-Film Transistors from Electrochemically Exfoliated In_2_Se_3_ Nanosheets

**DOI:** 10.3390/mi13060956

**Published:** 2022-06-16

**Authors:** Xiangxiang Gao, Hai-Yang Liu, Jincheng Zhang, Jian Zhu, Jingjing Chang, Yue Hao

**Affiliations:** 1 Advanced Interdisciplinary Research Center for Flexible Electronics, Xidian University, Xi’an 710071, China; gaoxiangxiang@xidian.edu.cn; 2 National Institute for Advanced Materials, School of Materials Science and Engineering, Nankai University, Tianjin 300350, China; haiyang-l@mail.nankai.edu.cn; 3 State Key Discipline Laboratory of Wide Band Gap Semiconductor Technology, School of Microelectronics, Xidian University, Xi’an 710071, China; jchzhang@xidian.edu.cn (J.Z.); yhao@xidian.edu.cn (Y.H.)

**Keywords:** two dimensional semiconductors, layer-by-layer assembly, field-effect transistors

## Abstract

The wafer-scale fabrication of two-dimensional (2D) semiconductor thin films is the key to the preparation of large-area electronic devices. Although chemical vapor deposition (CVD) solves this problem to a certain extent, complex processes are required to realize the transfer of thin films from the growth substrate to the device substrate, not to mention its harsh reaction conditions. The solution-based synthesis and assembly of 2D semiconductors could realize the large-scale preparation of 2D semiconductor thin films economically. In this work, indium selenide (In_2_Se_3_) nanosheets with uniform sizes and thicknesses were prepared by the electrochemical intercalation of quaternary ammonium ions into bulk crystals. Layer-by-layer (LbL) assembly was used to fabricate scalable and uniform In_2_Se_3_ thin films by coordinating In_2_Se_3_ with poly(diallyldimethylammonium chloride) (PDDA). Field-effect transistors (FETs) made from a single In_2_Se_3_ flake and In_2_Se_3_ thin films showed mobilities of 12.8 cm^2^·V^−1^·s^−1^ and 0.4 cm^2^·V^−1^·s^−1^, respectively, and on/off ratios of >10^3^. The solution self-assembled In_2_Se_3_ thin films enriches the research on wafer-scale 2D semiconductor thin films for electronics and optoelectronics and has broad prospects in high-performance and large-area flexible electronics.

## 1. Introduction

Two dimensional semiconductors have promoted the rapid development of electronics and optoelectronic devices due to their excellent charge transport properties and mechanical character [1,2]. The wafer-scale fabrication of well-ordered and uniform 2D semiconductor thin films are indispensable for large-area electronics and crucial to the practical application and development of 2D semiconductors [3,4,5]. High-quality 2D semiconductor thin films can be grown by chemical vapor deposition (CVD) [5,6,7]. However, the reaction conditions are stringent, and arduous transferring procedures are required to realize the transfer of thin films from the growth substrates to the targeted substrates. The transfer process is not only cumbersome and time-consuming, but it may also cause irreversible damage to the semiconductor device performance and reduce device yield.

Alternatively, uniform 2D semiconductor thin films can be economically prepared by a solution method from solution-processed 2D semiconductors colloidal inks [8,9]. Solution-processable 2D semiconductor electronics is an emerging research area, and substantial progress has been made. It has been reported that the electrochemical intercalation of quaternary ammonium ions is powerful in preparing stable 2D semiconductor inks such as graphite, black phosphorus, MoS_2_, In_2_Se_3_, and NbSe_2_ [10,11,12,13,14]. Their thin films have a wide range of applications in the fields of superconductors, field-effect transistors (FETs), photodetectors, and spin electronics. The spin-coated and LbL-assembled MoS_2_ thin films from electrochemically exfoliated MoS_2_ nanosheets used as FETs channels show mobilities of ≈10 cm^2^·V^−^^1^·s^−1^ and on/off ratios of >10^5^ [13,15]. Such device performance is superior to other solution-processed 2D semiconductor thin-film devices. The vacuum-filtrated In_2_Se_3_ thin films with thicknesses of 10 µm show ultrafast response, with rise and decay times of 41 and 39 ms, respectively, and efficient photoresponsivity (1 mA W^−1^) [16]. However, conventional, solution-based, thin-film deposition approaches confront the problems of uncontrollable film thickness, uneven deposition, and the coffee ring effect.

In this work, we propose LbL assembly as an effective method of fabricating scalable 2D thin films from electrochemically exfoliated nanosheets. LbL assembly is based on the alternating assembly of two species with complementary interactions (such as electrostatic attraction, hydrophobic interactions, or hydrogen bonds) and can prepare thin films, patterns, and heterostructures on any substrate. Various low-dimensional electronic nanofilms, including gold nanoparticles, single-walled carbon nanotubes, boronnitride, clay nanosheets, and MoS_2_, have been successfully assembled by LbL assembly with precise thickness control [15,17,18,19,20,21]. Here, we assembled uniform In_2_Se_3_ thin films by electrostatic adsorption between poly(diallyldimethylammonium chloride) (PDDA) and electrochemically exfoliated In_2_Se_3_. The single In_2_Se_3_ flake and LbL-assembled In_2_Se_3_ thin films, serving as active channel materials in FETs, possessed excellent device performance. The mobility and on/off ratio of the LbL-assembled In_2_Se_3_ thin films were even better than the CVD-grown In_2_Se_3_ thin film, showing the robustness of solution-processed electronics.

## 2. Materials and Methods

### 2.1. Materials

Bulk In_2_Se_3_ was purchased from Six Carbon Technology (Shenzhen, China). Tetraheptylammonium bromide (THAB) was purchased from Aladdin (Shanghai, China) Polyvinyl pyrrolidone (PVP) was purchased from Energy Chemical (Shanghai, China). Poly(diallyldimethylammonium chloride) (PDDA) was purchased from Sigma-Aldrich (Shanghai, China). Polyurethane was purchased from Yantai Wanhua Polyurethane Co., Ltd. (Yantai, China). Cr and Au metals were purchased from Vnano Vacuum Technology Co., Ltd. (Beijing, China). Carbon rod was purchased from Tianjin Aida Hengsheng Technology Development Co., Ltd. (Tianjin, China). Other solutions, including Acetonitrile, N,N–Dimethylformamide (DMF), acetone, and ethanol and isopropyl alcohol were purchased from Tianjin Chemical Reagent Company (Tianjin, China). Ultrapure water (20 MΩ. cm^−1^) was prepared by a Sartorius Arium pro UF system made by sartorius (Göttingen, Germany).

### 2.2. Synthesis of In_2_Se_3_ Nanosheets

In_2_Se_3_ nanosheets were synthesized by the electrochemical intercalation of quaternary ammonium ions. The electrochemical intercalation was performed in a 5 mg·mL^–1^ tetraheptylammonium bromide (THAB) acetonitrile solution, with the bulk In_2_Se_3_ and carbon rod serving as cathode and anode, respectively. The intercalation voltage was 8 V. After the intercalation, the THAB-intercalated In_2_Se_3_ was collected and sonicated in a 0.2 M polyvinyl pyrrolidone (PVP) solution (PVP: molecular weight of about 10,000) for 30 min to form a brown dispersion of In_2_Se_3_ nanosheets. The In_2_Se_3_ dispersion was subsequently centrifuged and washed with DMF several times to remove excessive PVP. The final In_2_Se_3_ dispersion was centrifuged at 1000 rpm for 5 min, and precipitates were discarded. The supernatant was concentrated in DMF for characterization and thin-film assembly.

### 2.3. Fabrication of In_2_Se_3_ Thin Films

The In_2_Se_3_ thin films were assembled by LbL assembly. Before LbL assembly, the SiO_2_/Si substrates were pre-cleaned with acetone as well as ethanol and isopropyl alcohol. The substrates were treated with oxygen plasma at 100 W for 5 min to produce a superwetting surface. The substrates were firstly immersed in a positively charged PDDA solution (0.1 wt %) for 2 min to deposit single-layer PDDA chains and were then rinsed by ultrapure water and gently dried with the use of an air gun. The substrates attached with PDDA chains were then immersed in negatively charged In_2_Se_3_ dispersion for 5 min, and In_2_Se_3_ nanosheets were assembled in order on the substrates by the electrostatic interaction between the PDDA and In_2_Se_3_. Finally, the substrates were rinsed by ultrapure water to remove the loosely attached In_2_Se_3_ nanosheets and were dried by air gun. The first cycle of the LbL assembly of the In_2_Se_3_ thin film was then completed. It is worth noting that the concentration of the In_2_Se_3_ dispersion was monitored by optical absorbance in order to assemble high-quality thin films. The single-layer, assembled In_2_Se_3_ thin films were dense when the characteristic absorbance at 450 nm was about 0.6 after the In_2_Se_3_ inks were diluted 500 times.

### 2.4. Fabrication of In_2_Se_3_ FETs

The channels of the FETs were fabricated by nanofiber masks. Aligned polyurethane nanofibers, whose diameters were maintained at ~500 nm, were printed on the substrates covered by In_2_Se_3_ single flakes or thin films. Subsequently, metal coatings (5 nm/45 nm Cr/Au) with a 200 × 200 μm metal mask were deposited by thermal evaporation to create source and drain electrodes. Finally, the SiO_2_/Si substrates were immersed in the DMF solvent for 30 min and sonicated for 5 min to remove the polyurethane fiber, and the channel of the FET devices could be successfully prepared. In order to improve the contact between the electrode and the In_2_Se_3_, the devices were annealed at 200 °C in vacuum for 2 h before the test.

## 3. Results

### 3.1. Synthesis and Characterizations of In_2_Se_3_ Nanosheets

**Figure 1:**
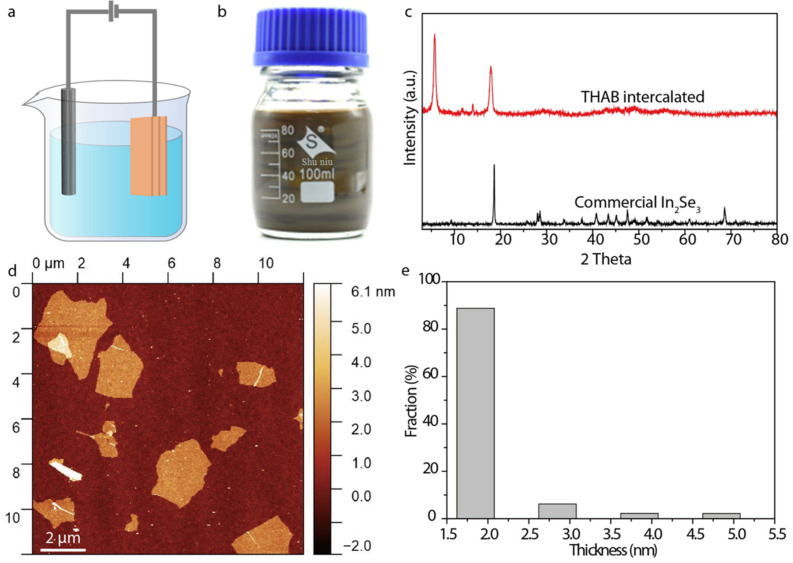
Synthesis and characterizations of In_2_Se_3_ nanosheets. (**a**) Schematic of the electrochemical exfoliation of the layered In_2_Se_3_ into nanosheets. (**b**) Photograph of In_2_Se_3_ dispersion in DMF. (**c**) XRD patterns of the THAB-intercalated bulk In_2_Se_3_ compared with commercial In_2_Se_3_. (**d**) Atomic force microscopy (AFM) image of In_2_Se_3_ nanosheets. (**e**) Thickness distribution of In_2_Se_3_ nanosheets.

**Figure 2:**
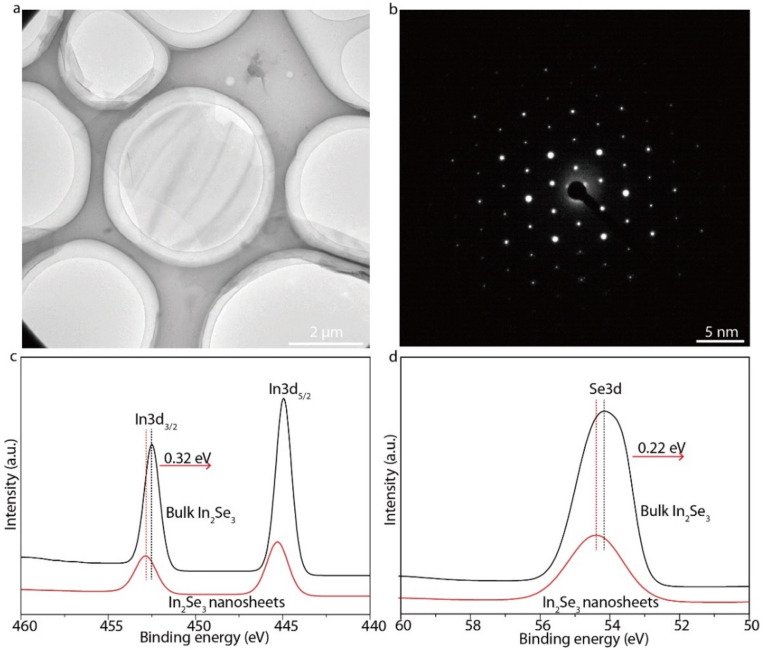
Characterizations of In_2_Se_3_ nanosheets. (**a**) TEM image of In_2_Se_3_ nanosheets. (**b**) Selected-area electron diffraction image of an In_2_Se_3_ nanosheet. (**c**) Comparison of In 3d binding energies of electrochemically intercalated In_2_Se_3_ nanosheets and bulk In_2_Se_3_. (**d**) Comparison of Se 3d binding energies of electrochemically intercalated In_2_Se_3_ nanosheets and bulk In_2_Se_3_.

In_2_Se_3_ nanosheets were synthesized by the electrochemical intercalation of quaternary ammonium ions, as shown in Figure 1a. The electrochemical intercalation was performed in a 5 mg·mL^–1^ tetraheptylammonium bromide (THAB) acetonitrile solution, with the bulk In_2_Se_3_ and the carbon rod serving as cathode and anode, respectively. Driven by external voltage, the positively charged THA^+^ ions were inserted into the bulk In_2_Se_3_ and became fluffy and fell off from the cathode. The THAB-intercalated In_2_Se_3_ were collected and sonicated in a 0.2 M polyvinyl pyrrolidone (PVP) solution (PVP: molecular weight of about 10,000) for 30 min to form a brown dispersion of In_2_Se_3_ nanosheets (Figure 1b). There is a new XRD peak at a diffraction angle of about 6 in the THAB-intercalated In_2_Se_3_, indicating that the interlayer spacing of the THAB-intercalated In_2_Se_3_ increased from 9.7 Å to 17 Å and further proved the successful insertion of the THA^+^ ions (Figure 1c). PVP acts as a surfactant to stabilize the In_2_Se_3_ nanosheet solution and prevent the agglomeration and sedimentation. The excessive PVP was removed by repeatedly washing with N,N-Dimethylformamide (DMF). The final In_2_Se_3_ dispersion was centrifuged at 1000 rpm. for 3 min to sort the nanosheets. The sediments containing unexfoliated layered crystallites were discarded. The supernatant was concentrated in DMF for characterization and thin-film assembly. AFM showed that the exfoliated In_2_Se_3_ nanosheets had micron-level lateral dimensions (Figure 1d). A total of 90% of the In_2_Se_3_ nanosheets have thicknesses of 2.2 nm, further confirming the few-layer nature of the In_2_Se_3_ nanosheets and the uniformity of the thicknesses (Figure 1e). The lamellar structure of the In_2_Se_3_ nanosheet was verified by a transmission electron microscopy (TEM) image (Figure 2a), and the selected area electron diffraction (SAED) patterns indicated the single crystalline characteristics of the In_2_Se_3_ nanosheet (Figure 2b). The In 3d and Se 3d binding energy peaks of the electrochemically intercalated In_2_Se_3_ shifted to higher values as compared with the bulk In_2_Se_3_ due to the n-type doping induced by the insertion of the THA^+^ ions (Figure 2c,d).

### 3.2. LbL Assembled In_2_Se_3_ Thin Films

**Figure 3:**
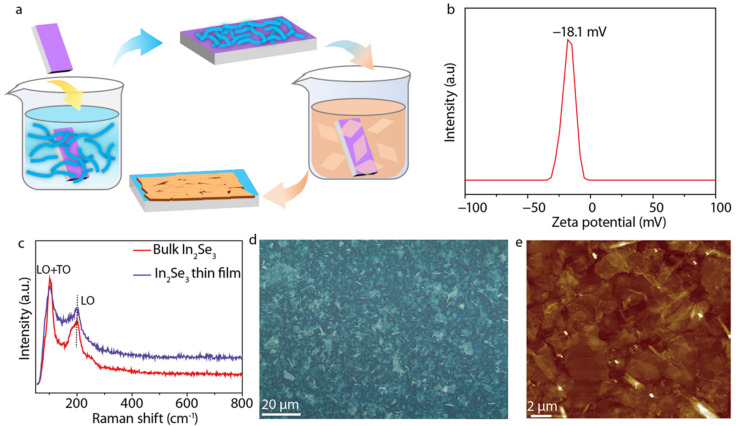
LbL assembly of In_2_Se_3_ thin films. (**a**) Schematic of the LbL assembly process. (**b**) Zeta potential of In_2_Se_3_ nanosheets dispersed in deionized water. (**c**) Raman spectra of In_2_Se_3_ thin films (purple) compared with bulk In_2_Se_3_ (red). (**d**) Optical microscope image of LbL-assembled In_2_Se_3_ thin films. (**e**) AFM image of LbL-assembled In_2_Se_3_ thin films.

**Figure 4:**
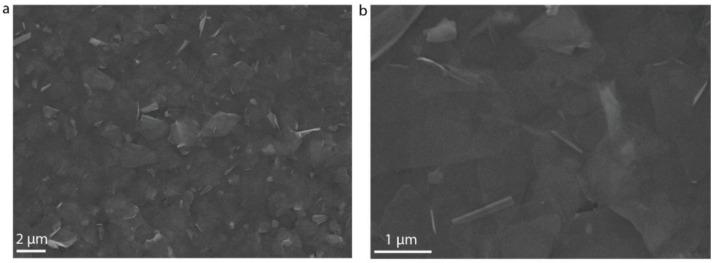
SEM image of LbL-assembled In_2_Se_3_ thin films at different magnifications. (**a**) SEM image of LbL-assembled In_2_Se_3_ thin films with 2 μm scale bar. (**b**) SEM image of LbL-assembled In_2_Se_3_ thin films with 1 μm scale bar.

**Figure 5:**
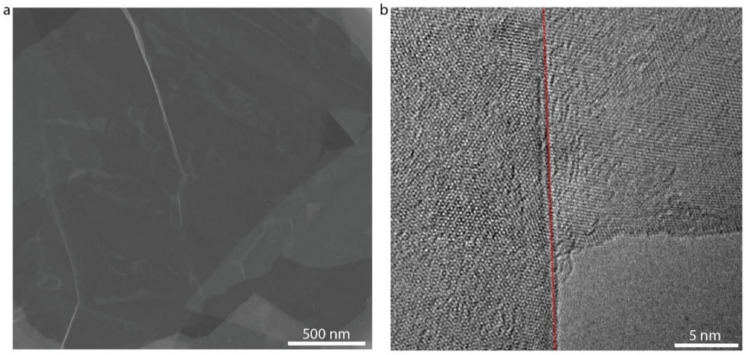
TEM image of LbL-assembled In_2_Se_3_ thin films. (**a**) Low-resolution TEM image of the LbL-assembled In_2_Se_3_ thin films. (**b**) High-resolution TEM image of the contact region between lateral nanosheets (The red dotted line is the edge of a nanosheet. The right shows the lattice of a single nanosheet, and the left shows the lattice of stacked nanosheets).

Well-ordered and uniform 2D semiconductor thin films are of vital importance to device performance. We chose LbL assembly to fabricate In_2_Se_3_ thin films by sequentially adsorbing the PDDA solution and the In_2_Se_3_ dispersion on SiO_2_/Si substrates through electrostatic interactions (Figure 3a). The zeta potential of the In_2_Se_3_ dispersion was found to be −18.1 mV (Figure 3b). The intercalation of tetraheptylammonium ions led to the injection of electrons into the In_2_Se_3_ crystal structures and the slightly negatively charged In_2_Se_3_ nanosheets [22]. Before LbL assembly, the SiO_2_/Si substrates were pre–cleaned with acetone as well as ethanol and isopropyl alcohol and then treated with oxygen plasma at 100 W for 5 min to produce a superwetting surface. The substrates were alternatively immersed in the PDDA solution (0.1 wt %) and the In_2_Se_3_ dispersion, with rinsing by ultrapure water and drying by air gun after each adsorption. The Raman characteristic peak originating from the A_1_(LO + TO) of the In_2_Se_3_ thin film were consistent with the bulk In_2_Se_3_, while the A_1_(LO) phonon mode of the In_2_Se_3_ thin film exhibited a small shift toward lower wavenumbers arising from the smaller vibration coherence length along the c-axis as a result of the weak van der Waals interaction (Figure 3c). The optical microscope image of the LbL-assembled In_2_Se_3_ thin film revealed that the nanosheets in a wide range of films were evenly stacked and assembled into homogeneous thin films (Figure 3d). From the local AFM and SEM image of the LbL-assembled In_2_Se_3_ thin film, we can deduce that the adjacent nanosheets were assembled on the substrate through broad-area, plane-to-plane Van der Waals contacts (Figure 3e and Figure 4). The TEM images of the LbL-assembled In_2_Se_3_ thin film show that the adjacent nanosheets are stacked tightly together with mixed crystalline lattices on the boundaries and further demonstrate decent interfaces (Figure 5). The number of in-plane grain boundaries in the LbL-assembled 2D semiconductor thin films were greatly reduced and will significantly improve charge transport performance.

### 3.3. Performance of FETs from Electrochemically Exfoliated In_2_Se_3_ Nanosheets

**Figure 6:**
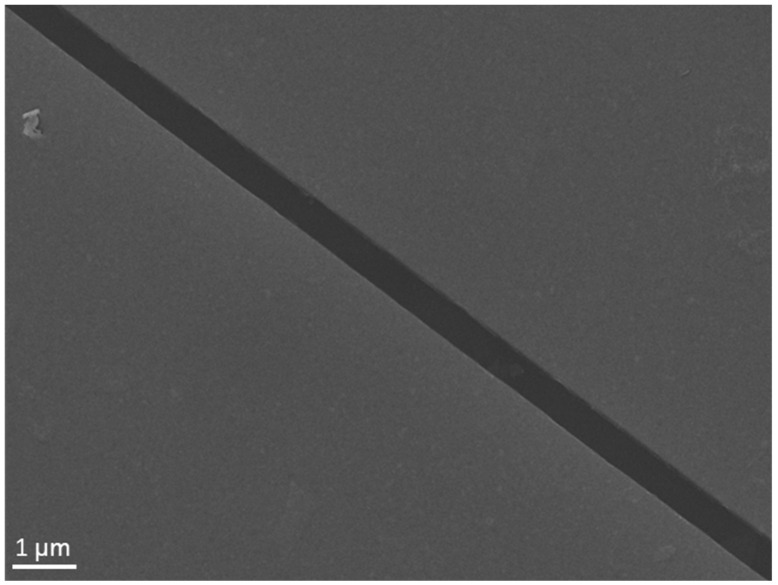
SEM image of the channel fabricated by nanofiber masks.

**Figure 7:**
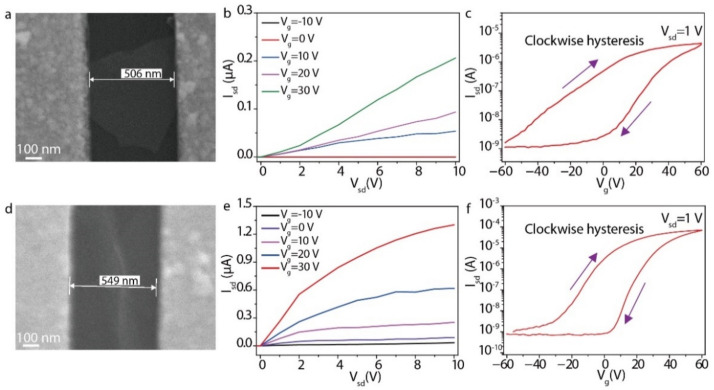
Transistor performance of In_2_Se_3_. (**a**) SEM image of the In_2_Se_3_ single-flake FET with a single In_2_Se_3_ nanosheet as the FET channel. (**b**) *I*_sd_–*V*_sd_ output characteristics of the In_2_Se_3_ single-flake FET. (**c**) *I*_sd_–*V*_g_ transfer characteristics of the In_2_Se_3_ single-flake FET. (**d**) SEM image of the In_2_Se_3_ thin-film FET channel. (**e**) *I*_sd_–*V*_sd_ output characteristics of the In_2_Se_3_ thin-film FET. (**f**) *I*_sd_–*V*_g_ transfer characteristics of the In_2_Se_3_ thin-film FET.

**Figure 8:**
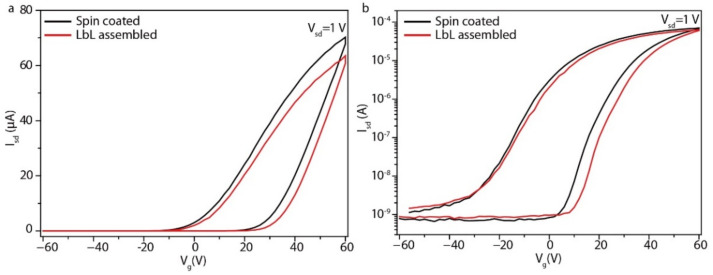
(**a**) *I*_sd_-*V*_sd_ transfer characteristics of the LbL-assembled and spin-coated In_2_Se_3_ thin film FETs on a linear scale. (**b**) *I*_sd_-*V*_sd_ transfer characteristics of the LbL-assembled and spin-coated In_2_Se_3_ thin film FETs on a logarithmic scale.

To investigate the electric properties of solution-processed In_2_Se_3_, we further prepared In_2_Se_3_ single-flake and In_2_Se_3_ thin-film FETs on a 300 nm SiO_2_/Si substrate. The channels of the FETs were fabricated by nanofiber masks (Figure 6) [23]. First, we used the diluted and concentrated In_2_Se_3_ dispersion to adsorb the sparse In_2_Se_3_ nanosheets and dense In_2_Se_3_ thin films on a pre-treated SiO_2_/Si substrate by LbL assembly. Then, polyurethane nanofibers were printed and metal electrodes were deposited in order to fabricate FET devices. Figure 7a shows the scanning electron microscope (SEM) image of the In_2_Se_3_ single-flake FET with a length of 506 nm and an average width of 600 nm. The In_2_Se_3_ single-flake is perfectly flat on the channel to ensure good contact between the electrode and the In_2_Se_3_ nanosheet. The *I*_sd_–*V*_sd_ output characteristics of the In_2_Se_3_ single-flake FET showed a linear trend, indicating ohmic contacts between the In_2_Se_3_ single-flake and electrode (Figure 7b). The forward and reverse *I*_sd_–*V*_g_ transfer characteristics of the In_2_Se_3_ single-flake FET showed a typical n-type behavior with an on/off ratio of 1.5 × 10^3^ at *V*_sd_ = 1 V (Figure 7c). The electron mobility of individual In_2_Se_3_ nanosheets can be calculated to be 12.8 cm^2^ V^−1^ s^−1^ from the linear−regime transfer characteristics using the following equation:

$$\mu =\frac{dI_{\mathrm{sd}}}{dV_{g}}\times \frac{L}{WC_{s}V_{\mathrm{sd}}}$$

where *L* and *W* are the channel length and width, and *C*_s_ is the areal capacitance of 300 nm SiO_2_/Si. The channel length and width of the In_2_Se_3_ thin film FET device are 549 nm and 200 μm, respectively (Figure 7d). The *I*_sd_–*V*_sd_ output characteristics of the In_2_Se_3_ thin film FET exhibited non-linear dependence on *V*_sd_ due to the pinch-off effect of the FET channel (Figure 7e). The electron mobility of In_2_Se_3_ thin films reached 0.2 cm^2^ V^−1^ s^−1^, with an on/off ratio of 7 × 10^4^ at *V*_sd_ = 1 V (Figure 7f). The carrier mobility of the In_2_Se_3_ single flake is much higher than that of the In_2_Se_3_ thin film due to the sheet-to-sheet contact resistance, and the device performance may average out in the percolating thin films. The observed clockwise hysteresis in the transfer characteristics of the In_2_Se_3_ single-flake and thin-film FETs was attributed to charge trapping and detrapping at the interface between the In_2_Se_3_ and the SiO_2_ (Figure 7c,f) [24]. To further understand the relative effects of PDDA, the spin-coated In_2_Se_3_ thin-film FETs were fabricated. The doping of the PDDA caused the positive shift of the threshold voltage and the lower maximum on-current as compared with the spin-coated In_2_Se_3_ thin-film FET (Figure 8a,b).

The performance of electrochemically exfoliated In_2_Se_3_ single-flake and thin-film FETs were comparable to those made from other methods in terms of mobilities and on/off ratios (Table 1) [25,26,27,28,29]. A comprehensive comparison is provided in Table 1. The electrochemically exfoliated In_2_Se_3_ single-flake FET performance was superior to some mechanically exfoliated In_2_Se_3_ flake FETs [27]. The electron mobilities and on/off ratios of the LbL-assembled In_2_Se_3_ thin film was close to those of the spin-coated In_2_Se_3_ thin films and CVD-grown In_2_Se_3_ thin films [12,28]. The outstanding device performances of both single flakes and thin films are attributed to the high-quality nanosheets with uniform sizes and thicknesses prepared by electrochemical intercalation.

## 4. Conclusions

In conclusion, we prepared high-quality In_2_Se_3_ nanosheets through an electrochemical intercalation approach. Homogeneous In_2_Se_3_ thin films were assembled by the alternate adsorption of PDDA and a nanosheet solution, driven by electrostatic attraction. FETs from solution-processed In_2_Se_3_ single flakes and thin films showed satisfying performance and were comparable to those from CVD-grown In_2_Se_3_ thin films, mechanically exfoliated In_2_Se_3_ flakes, and spin-coated In_2_Se_3_ thin films. LbL-assembled 2D semiconductor thin films are promising candidates for emerging large-area, flexible, and wearable electronic applications.

## Figures and Tables

**Table 1:** Comparison of In_2_Se_3_ FET performance with different preparation methods.

Preparation Methods	Mobility (cm^2^ V^−1^ s^−1^)	On/Off Ratio	Reference
Mechanically exfoliated In_2_Se_3_ flake	30	10^5^	[25]
Mechanically exfoliated In_2_Se_3_ flake	1.26	500	[27]
CVD-grown In_2_Se_3_ thin film	2.51 × 10^−3^	12	[26]
CVD-grown In_2_Se_3_ thin film	1	10^3^	[28]
PVD-grown p type In_2_Se_3_ flake	2.5	10^3^	[29]
Spin-coated In_2_Se_3_ thin film	0.2	10^5^	[12]
Electrochemically exfoliated In_2_Se_3_ flake	12.8	1.5 × 10^3^	This work
LbL-assembled In_2_Se_3_ thin film	0.4	7 × 10^4^	

## Data Availability

The data used to support the findings of this study are available from the corresponding author upon request.

## References

[1] Liu C., Chen H., Wang S., Liu Q., Jiang Y.-G., Zhang D.W., Liu M., Zhou P. (2020). Two-dimensional materials for next-generation computing technologies. Nat. Nanotechnol..

[2] Wang Q.H., Kalantar-Zadeh K., Kis A., Coleman J.N., Strano M.S. (2012). Electronics and optoelectronics of two-dimensional transition metal dichalcogenides. Nat. Nanotechnol..

[3] Lin Z., McCreary A., Briggs N., Subramanian S., Zhang K.H., Sun Y.F., Li X.F., Borys N.J., Yuan H.T., Fullerton-Shirey S.K. (2016). 2D materials advances: From large scale synthesis and controlled heterostructures to improved characterization techniques, defects and applications. 2D Mater..

[4] Chen X., Xie Y., Sheng Y., Tang H., Wang Z., Wang Y., Wang Y., Liao F., Ma J., Guo X. (2021). Wafer-scale functional circuits based on two dimensional semiconductors with fabrication optimized by machine learning. Nat. Commun..

[5] Li T., Guo W., Ma L., Li W., Yu Z., Han Z., Gao S., Liu L., Fan D., Wang Z. (2021). Epitaxial growth of wafer-scale molybdenum disulfide semiconductor single crystals on sapphire. Nat. Nanotechnol..

[6] Li N., Wang Q., Shen C., Wei Z., Yu H., Zhao J., Lu X., Wang G., He C., Xie L. (2020). Large-scale flexible and transparent electronics based on monolayer molybdenum disulfide field-effect transistors. Nat. Electron..

[7] Zhou J., Lin J., Huang X., Zhou Y., Chen Y., Xia J., Wang H., Xie Y., Yu H., Lei J. (2018). A library of atomically thin metal chalcogenides. Nature.

[8] Gao X., Bian G., Zhu J. (2019). Electronics from solution-processed 2D semiconductors. J. Mater. Chem. C.

[9] Lin Z., Huang Y., Duan X. (2019). Van der Waals thin-film electronics. Nat. Electron..

[10] Zhang Y., Xu Y. (2019). Simultaneous electrochemical dual-electrode exfoliation of graphite toward scalable production of high-quality graphene. Adv. Funct. Mater..

[11] Wang N., Mao N., Wang Z., Yang X., Zhou X., Liu H., Qiao S., Lei X., Wang J., Xu H. (2020). Electrochemical delamination of ultralarge few-layer black phosphorus with a hydrogen-free intercalation mechanism. Adv. Mater..

[12] Lin Z., Wan Z., Song F., Huang B., Jia C., Qian Q., Kang J.S., Wu Y., Yan X., Peng L. (2021). High-yield exfoliation of 2D semiconductor monolayers and reassembly of organic/inorganic artificial superlattices. Chem.

[13] Lin Z., Liu Y., Halim U., Ding M., Liu Y., Wang Y., Jia C., Chen P., Duan X., Wang C. (2018). Solution-processable 2D semiconductors for high-performance large-area electronics. Nature.

[14] Li J., Song P., Zhao J., Vaklinova K., Zhao X., Li Z., Qiu Z., Wang Z., Lin L., Zhao M. (2020). Printable two-dimensional superconducting monolayers. Nat. Mater..

[15] Gao X., Yin J., Bian G., Liu H.-Y., Wang C.-P., Pang X.-X., Zhu J. (2021). High-mobility patternable MoS_2_ percolating nanofilms. Nano Res..

[16] Shi H., Li M., Shaygan Nia A., Wang M., Park S., Zhang Z., Lohe M.R., Yang S., Feng X. (2020). Ultrafast electrochemical synthesis of defect-free In_2_Se_3_ flakes for large-area optoelectronics. Adv. Mater..

[17] Kim Y., Zhu J., Yeom B., Di Prima M., Su X., Kim J.-G., Yoo S.J., Uher C., Kotov N.A. (2013). Stretchable nanoparticle conductors with self-organized conductive pathways. Nature.

[18] Wu J., Antaris A., Gong M., Dai H. (2014). Top-down patterning and self-assembly for regular arrays of semiconducting single-walled carbon nanotubes. Adv. Mater..

[19] Zhu J., Liu X., Geier M.L., McMorrow J.J., Jariwala D., Beck M.E., Huang W., Marks T.J., Hersam M.C. (2016). Layer-by-Layer assembled 2D montmorillonite dielectrics for solution-processed electronics. Adv. Mater..

[20] Zhu J., Kang J., Kang J., Jariwala D., Wood J.D., Seo J.-W.T., Chen K.-S., Marks T.J., Hersam M.C. (2015). Solution-processed dielectrics based on thickness-sorted two-dimensional hexagonal boron nitride nanosheets. Nano Lett..

[21] Richardson J.J., Björnmalm M., Caruso F. (2015). Technology-driven layer-by-layer assembly of nanofilms. Science.

[22] Wang C., He Q., Halim U., Liu Y., Zhu E., Lin Z., Xiao H., Duan X., Feng Z., Cheng R. (2018). Monolayer atomic crystal molecular superlattices. Nature.

[23] Liu H.-Y., Yin J., Gao X., Zhao S., Bian G., Li J., Wang C.-P., Zhu J. (2021). Scalable submicron channel fabrication by suspended nanofiber lithography for short-channel field-effect transistors. Adv. Funct. Mater..

[24] Guo Y., Wei X., Shu J., Liu B., Yin J., Guan C., Han Y., Gao S., Chen Q. (2015). Charge trapping at the MoS_2_-SiO_2_ interface and its effects on the characteristics of MoS2 metal-oxide-semiconductor field effect transistors. Appl. Phys. Lett..

[25] Island J.O., Blanter S.I., Buscema M., van der Zant H.S.J., Castellanos-Gomez A. (2015). Gate controlled photocurrent generation mechanisms in high-gain In_2_Se_3_ phototransistors. Nano Lett..

[26] Mukherjee S., Dutta D., Mohapatra P.K., Dezanashvili L., Ismach A., Koren E. (2020). Scalable integration of coplanar heterojunction monolithic devices on two-dimensional In_2_Se_3_. ACS Nano.

[27] Feng W., Gao F., Hu Y., Dai M., Liu H., Wang L., Hu P. (2018). Phase-engineering-driven enhanced electronic and optoelectronic performance of multilayer In_2_Se_3_ nanosheets. ACS Appl. Mater. Interfaces.

[28] Feng W., Gao F., Hu Y., Dai M., Li H., Wang L., Hu P. (2018). High-performance and flexible photodetectors based on chemical vapor deposition grown two-dimensional In_2_Se_3_ nanosheets. Nanotechnology.

[29] Zhou J., Zeng Q., Lv D., Sun L., Niu L., Fu W., Liu F., Shen Z., Jin C., Liu Z. (2015). Controlled synthesis of high-quality monolayered α-In_2_Se_3_ via physical vapor deposition. Nano Lett..

